# Should my child play contact sports?

**DOI:** 10.1111/head.70141

**Published:** 2026-06-05

**Authors:** Carlyn Patterson Gentile

**Affiliations:** ^1^ Children's Hospital of Philadelphia Philadelphia Pennsylvania USA; ^2^ Perelman School of Medicine at the University of Pennsylvania Philadelphia Pennsylvania USA

**Keywords:** pediatric, post‐traumatic headache, sports‐related concussion

As a pediatric headache specialist with a practice focused on managing post‐traumatic headache and as the parent of two school‐age children, should my child play contact sports is a question I face frequently in my professional and personal life. It is also a question that does not have a straightforward one‐size‐fits‐all answer. This decision should be individualized, based on shared decision‐making between the caregivers and child, and supported by healthcare professionals to weigh the potential risks, benefits, and uncertainties.

Participation in organized sports undoubtably offers many benefits important for child development. It guarantees physical activity that can improve cardiovascular endurance and physical fitness, improve sleep, and provide a myriad other positive downstream effects.^1^, ^2^, ^3^ Participation in youth sports occurs at an important period of habit formation that can have long‐term health benefits. Additionally, sports offer an environment that improves mental health outcomes; youths receive social support that develops leadership skills, fosters a sense of belonging as part of a team, and encourages a strong work ethic.^3^, ^4^, ^5^, ^6^ For these reasons, Healthy People 2030 is a health initiative set by the US Department of Health and Human Services aimed at increasing youth sports participation to 63.3% by 2030. Youth sports have declined in recent years to as low as 50.7% in 2020–2021.^7^ Models project that increasing youth sports participation would reduce obesity prevalence and, by extension, weight‐related disease; improve quality of life; and reduce anxiety and depression symptoms.^7^ Indeed, the positive impact of organized sports on health in youth is multifaceted.

However, sports is not without risk. Sport‐related injury is a common cause of concussion in preteens and adolescents.^8^ The risk of concussion varies by sport. In noncontact sports, there is a low likelihood of coming into bodily contact with another participant (e.g., swimming, cross‐country running). By comparison, in contact sports (e.g., soccer, basketball), there is a high likelihood of participants coming into bodily contact with one another, but it may not be forceful or intentional. In collision sports (e.g., American football, rugby), play requires high‐intensity, intentional contact. Concussion results in multiple symptoms (e.g., headache, dizziness, sensory sensitivity, cognitive symptoms, emotional changes) that resolve for most in 2–4 weeks, whereas others experience prolonged symptoms.^9^ Potential risk factors for experiencing prolonged recovery include prior history of concussion (particularly with recovery taking >1 week), preexisting medical conditions (i.e., migraine, anxiety, depression, attention‐deficit/hyperactivity disorder), high symptom burden acutely following concussion, female sex, adolescent age, and delayed reporting.^10^, ^11^, ^12^

In addition to clinical syndrome concussion, contact and collision sports can also expose an individual to repetitive head impacts, which has raised concern for long‐term neurologic health. The most severe outcome, chronic traumatic encephalopathy (CTE) has entered the public Zeitgeist. CTE is not a clinical syndrome but rather is defined by a buildup of tau protein that is seen in individuals with a history of repetitive head trauma. CTE is purely defined by this pathological finding on autopsy. However, individuals with CTE pathological findings have been reported to experience progressive, degenerative neurological symptoms including cognitive and behavioral changes. In a study of 152 symptomatic contact sports athletes, 41% had evidence of CTE on brain autopsy.^13^ Nearly all cases were mild. The most common sports played was American football, but other sports included ice hockey, rugby, wrestling, and soccer. Those with CTE had played significantly more years of sport. It is important to note the limitations of this study. It only included athletes who died prior to age 30 years and whose families were concerned about CTE; a few had additional risk factors for repetitive head trauma (e.g., military service). This finding requires a deeper understanding of the risk of repetitive head trauma in amateur sport, though it should be emphasized that this was a highly selective sample that does not address the prevalence of CTE.

Studies assessing long‐term outcomes in those who participated in youth and collegiate contact/collision sports have largely not found worse long‐term neurological outcomes, though studies have reported higher prevalence of neurodegenerative diseases in professional contact sports athletes.^14^, ^15^ The question remains as to why do some people develop prolonged sequelae from repetitive head trauma, while most do not. Risk is likely multifactorial and may include genetic risk factors, age of first impact, frequency of impacts, and longer duration of exposure, among others. It remains unclear to what extent repeated concussion versus frequent subconcussive repetitive head trauma play a role. There are currently no definitions of mechanical force linked to increased risk of poor neurological outcome. Further study is needed to address these open questions in order to be able to define individualized risk for our patients.

The consensus statement on concussion in sports reflects the lack of clarity and broad perspectives on the risk of repetitive head trauma.^15^ As healthcare providers, we are left to help our patients and their caregivers navigate through this uncertainty. Multiple factors should be considered when counseling a child and their family.^15^ This includes the child's interests because organized sports is one way to gain multiple physical and social benefits but may not be the best way for every child. For other children, contact sports may provide an outlet that noncontact sports do not. It is also important to be aware of the context, including what extracurricular activities are available, family values, cultural factors, and financial implications, because sports can be a pathway to higher education for some athletes. Another consideration is medical comorbidities, again weighing the potential risks and benefits. For instance, those with a family or personal history of migraine may be at risk for prolonged recovery,^16^ but important features of migraine management include regular exercise, sleep, and stress management, which organized sports can support. Concussion history is also important. Based on current evidence, there is no defined set number of concussions that necessitates retirement from sport.^15^ The mechanism of injury (in vs. out of sport), longer recovery, and/or less force is needed to elicit concussion symptoms, and the athlete and their caregivers' readiness/apprehension for return to sports should be considered. Such decisions are best made, wherever possible, with multidisciplinary clinical evaluation,^15^ ideally including longitudinal follow‐up with a sports medicine physician and/or neurologist specializing in concussion. Because sports can be core to an athlete's identity, it is important to discuss transition out of a high level of competition, regardless of the circumstances of their retirement.

On a systems level, efforts are underway to reduce concussion risk and improve safety in sport. In recent years, there has been a threefold increase in studies examining the effectiveness of sport‐related concussion prevention strategies.^17^ There have also been success stories. A policy disallowing body checking in child and adolescent ice hockey reduced the rate of game‐related concussions. In American football, limiting the number, duration, and intensity of contact practices and restricting collision reduced practice‐related concussion and head impact rates. The use of personal protective equipment has been found to be beneficial in some sports. Mouth guards have been found to reduce concussion incidence in ice hockey,^18^ and on‐field neuromuscular training warm‐up programs have been associated with a reduction in concussion rates in rugby.^19^ However, other protective equipment (e.g., Q‐collars, Guardian Caps) has no or low‐quality evidence to support its use in youth sports.^20^, ^21^ Furthermore, the utility of different forms of protective equipment may differ by sport; thus, the use of protective gear should be tailored to the specific sport.^17^ Additionally, having a plan for managing concussion, including mandatory removal from play following suspected head injury, required clearance from a medical professional, and concussion education for coaches, players, and parents, reduces concussion recurrence.^17^ It is important that as new safety procedures are enacted, the effects on concussion rates are observed in all settings, including practice and competition.

In summary, there are myriad benefits of youth sports but also potential risks. Counseling requires an individualized approach (Table 1). There is substantial heterogeneity in concussion outcomes, and currently, our ability to predict specific risk is limited. Further research is needed to better understand the long‐term effects of concussion and repetitive head impacts to inform clinical decision making.

**TABLE 1:** Framework for clinician‐guided shared decision‐making on youth contact sports.

Shared decision‐making	Considerations	
Key points Open communication between healthcare provider/care team, the child, their caregiver(s), and other groups (e.g., school, sports teams) as appropriate Consider all factors, weigh risks and benefit Longitudinal follow‐up, if needed Heterogeneous recovery from concussion with limited ability to predict individual risk/outcomes Based on available evidence, no set number of concussions necessitate retirement from sport	The child *Individual factors*	Interests History of concussion and recovery, particularly sport‐related Preexisting conditions (e.g., migraine, ADHD) Psychological (stress management, identity, social circle)
	The broader environment *Contextual factors*	Cultural norms Family values Economic factors Ethical factors Availability of sport sports being played
	The uncertainty *Limited evidence base for clinical decision‐making*	Limited ability to predict individual risk, Prolonged post‐concussion symptoms Long‐term sequelae from repetitive head trauma Child/caregiver(s) readiness/apprehension to potential risk and uncertainty

Abbreviations: ADHD, attention‐deficit/hyperactivity disorder.

*Source*: Adapted from recommendations from Consensus Statement on Concussion in Sport: The 6th International Conference on Concussion in Sport–Amsterdam, October 2022.^15^

## AUTHOR CONTRIBUTIONS

**Carlyn Patterson Gentile:** Conceptualization; writing – original draft.

## FUNDING INFORMATION

This work was funded by the National Institutes of Health (NIH)/National Institute of Neurological Disorders and Stroke (NINDS), grant K23NS124986 to CPG.

## CONFLICT OF INTEREST STATEMENT

**Carlyn Patterson Gentile** is currently funded by the National Institutes of Health/National Institute of Neurological Disorders and Stroke (K23NS124986).
